# Causal relationships between immune cells, inflammatory cytokines, and pertussis: Bidirectional 2-sample Mendelian randomization study and mediation analysis

**DOI:** 10.1097/MD.0000000000040712

**Published:** 2024-11-29

**Authors:** Fu-Qing Lu, Hui-Mei Feng, Ji-Gan Wang, Kun-Ling Song

**Affiliations:** a Department of Pediatrics, The People’s Hospital of Guangxi Zhuang Autonomous Region, Nanning, China; b Department of Pediatrics, Maternal and Child Health Hospital of Guangxi Zhuang Autonomous Region, Guangxi Clinical Research Center for Pediatric Diseases, Nanning, China.

**Keywords:** GWAS, immune cells, inflammatory cytokines, mediation analysis, Mendelian randomization, pertussis

## Abstract

Studies have shown that immune cells play an important role in the occurrence and development of pertussis, but the specific causal relationships are yet to be determined. Additionally, inflammatory cytokines, as regulators of immune responses, may mediate the relationship between immune cells and pertussis, and the specific mechanisms involved require further exploration. This study utilizes data from multiple large-scale genome-wide association studies, covering 731 types of immune cells and 91 types of inflammatory cytokines. The bidirectional 2-sample Mendelian randomization (MR) method is employed, with inverse-variance weighted as the main statistical approach, to assess the causal relationships between immune cells, inflammatory cytokines, and pertussis. Furthermore, a 2-step MR method is used to investigate the mediating role of inflammatory cytokines in the effect of immune cells on pertussis. Our study results indicate that 11 types of immune cells have a protective effect against pertussis, with the strongest protection observed from CD25 on CD28+ CD4+ cells (OR = 0.3533, CI = 0.1636–0.7627, *P* = .008). Conversely, 19 types of immune cells are positively associated with the risk of pertussis, with the strongest correlation found in CD3− lymphocyte %lymphocyte (OR = 3.6613, CI = 1.5012–8.299, *P* = .0043). Additionally, 3 inflammatory cytokines – IL-4, IL-18R1, and FGF-21 – show a causal relationship with pertussis. Our mediation MR results indicate that inflammatory cytokines do not act as mediators in the relationship between immune cells and pertussis. This study suggests a causal relationship between immune cells and pertussis, while inflammatory cytokines do not appear to be mediating factors in the pathway from immune cells to pertussis.

## 1. Introduction

Pertussis, also known as whooping cough, is a highly contagious respiratory disease caused by Bordetella pertussis.^[1,2]^ Despite the significant global decline in pertussis incidence through vaccination, in recent years, reduced vaccine effectiveness, pathogen variation, and waning immunity have led to a resurgence of pertussis cases in certain regions.^[3]^ pertussis continues to pose a substantial public health burden worldwide.^[4,5]^ The high incidence and severe complications of pertussis in infants and young children have garnered extensive attention.^[6]^ However, the specific roles and interactions of immune cells and inflammatory cytokines in the pathogenesis of pertussis remain unclear in current research.

The immune system plays a crucial role in defending against pathogenic infections, with various immune cells and inflammatory cytokines playing important roles in regulating immune responses.^[7]^ While some studies suggest that certain immune cells may be associated with susceptibility to and severity of pertussis, their causal relationships have not been fully validated. Additionally, inflammatory cytokines, as regulators of immune responses, may mediate the link between immune cells and pertussis, but this hypothesis also lacks systematic research and validation.

To fill this knowledge gap, this study aims to systematically evaluate the impact of immune cells and inflammatory cytokines on pertussis through bidirectional 2-sample Mendelian randomization (MR) studies and mediation analysis. Using inverse-variance weighted (IVW) as the main statistical method, we first assess the direct causal effects of immune cells on pertussis. Subsequently, a 2-step MR approach is employed to investigate the mediating role of inflammatory cytokines in the effects of immune cells on pertussis. The study data encompass 731 types of immune cells and 91 types of inflammatory cytokines, providing a large-scale and comprehensive analytical foundation.

The results of this study will reveal the key roles of specific immune cells and inflammatory cytokines in the pathogenesis of pertussis and further clarify their causal relationships. By identifying protective and risk-related immune cells associated with pertussis, we aim to provide new potential targets for disease diagnosis and treatment. Additionally, exploring the mediating effects of inflammatory cytokines in the influence of immune cells on pertussis will aid in understanding more complex immune regulatory mechanisms and offer new research directions and clinical applications for developing immunomodulatory therapeutic strategies in the future.

Mendelian randomization uses genetic variation (typically single nucleotide polymorphisms [SNPs]) as IVs to estimate the causal relationship between exposure factors (e.g., lifestyle, environmental factors) and outcomes (e.g., diseases). Since genes are randomly assigned during fertilization and are not influenced by most environmental or behavioral factors, MR effectively avoids common confounding issues in traditional observational studies. Compared to general association studies, MR is closer to a randomized controlled trial, as it mimics “randomization” through the natural distribution of genes. This makes MR analysis more reliable in inferring causality rather than just identifying observational associations.^[8]^

Additionally, the application of bidirectional 2-sample MR allows us to explore causal relationships from both directions, not only assessing the effect of immune cells on pertussis but also detecting potential feedback effects of pertussis on immune cells. This bidirectional approach is more innovative than traditional unidirectional causal inference methods, revealing more complex biological interactions.

This study further innovatively introduces a 2-step MR method to explore whether inflammatory cytokines act as mediators in the influence of immune cells on pertussis. By assessing this mediation effect, the study not only reveals direct causal pathways but also delves into more complex immune regulatory mechanisms, providing theoretical support for future immunological intervention strategies for pertussis.

In summary, this study aims to systematically explore the causal relationship between immune cells and pertussis through the MR approach, while also evaluating the mediating role of inflammatory cytokines. Compared to the limitations of previous observational studies, the bidirectional MR and mediation effect analysis employed here can more accurately reveal the immunological mechanisms of pertussis, offering important new insights into the roles of immune cells and inflammatory cytokines in infectious diseases.

## 2. Method

### 2.1. Step 1: direct causal relationship between immune cells and pertussis

First, we evaluated the direct causal effect of various immune cells on pertussis. Using the 2-sample MR method, we inferred the causal relationship by examining the association between genetic variations in different immune cells and the incidence of pertussis.

**Figure 1.**
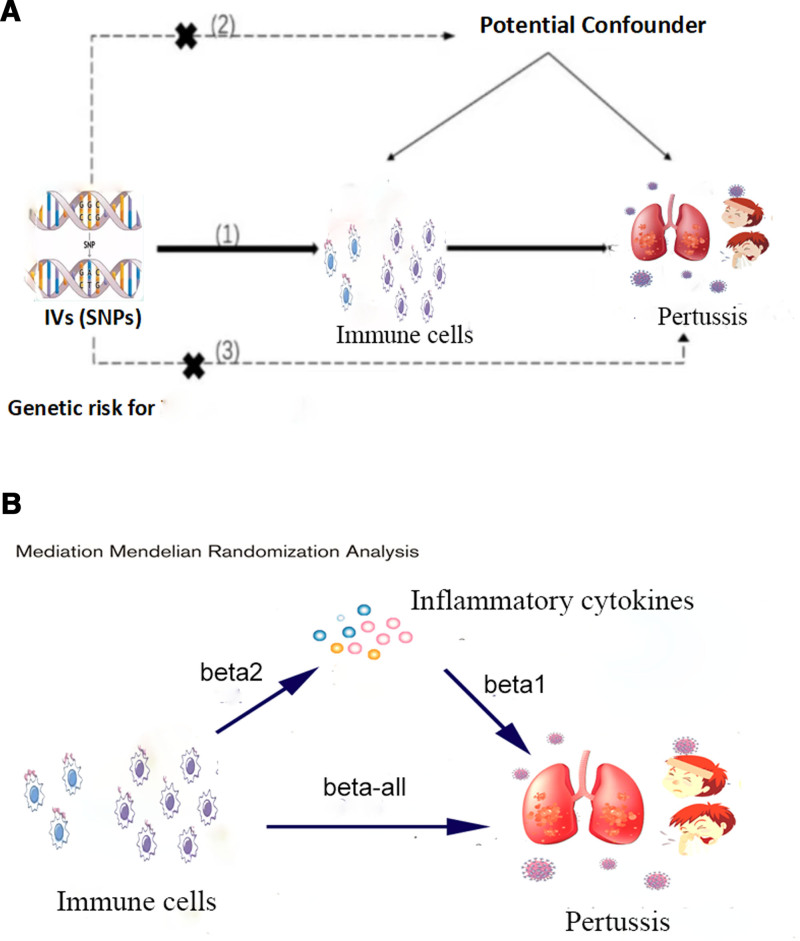
(A) Schematic diagram of the 2-sample Mendelian randomization analysis on the association between immune cells and pertussis. (B) The study design for the mediation of relationship between immune cells and pertussis by inflammatory cytokines.

In this study, we first performed a 2-sample bidirectional MR to examine the causal relationship between immune cells and pertussis. Procedures followed the latest STROBE-MR guidelines (Supplementary-STROBE-MR Checklist, Supplemental Digital Content, http://links.lww.com/MD/O70) and was conducted under the following 3 assumptions:^[9]^ instrumental variables (IVs) must be significantly associated with immune cells; IVs are not associated with other factors that could confound the relationship between exposure and outcome variables; IVs should influence outcomes solely via the immune cells (Fig. 1A).

### 2.2. Step 2: mediating role of inflammatory cytokines

The causal relationship between inflammatory cytokines (mediators) and pertussis (outcome) was evaluated using the 2-sample MR method to identify inflammatory cytokines with causal relationships, resulting in beta1. In the first step, immune cells with a causal relationship to the outcome were identified, and the 2-sample MR method was applied to assess their relationship with inflammatory cytokines, yielding beta2. Mediation analysis can only be conducted when a causal triangular relationship exists, meaning there is a causal relationship between exposure and outcome, between the mediator and the outcome, and between exposure and the mediator.

Mediation effect = beta2 * beta1, and mediation proportion = (beta2 * beta1)/ beta-all (Fig. 1B).

### 2.3. Data sources

The genome-wide association studies (GWAS) catalog provides publicly available summary statistics for 731 immune cells (with accession numbers ranging from GCST90001391 to GCST90002121).^[10]^ A comprehensive analysis was conducted on a total of 731 immunophenotypes, involving various types of data, such as absolute cell counts (AC), median fluorescence intensities which represented surface antigen levels, morphological parameters, and relative cell counts (RC). The initial GWAS on immune traits utilized data from 3757 individuals of European descent, with no identified overlap in cohorts.

Inflammatory cytokines (91 inflammatory factors, GCST90274758.tsv to GCST90274848.tsv) were derived from 14,824 individuals across 11 cohorts of European ancestry,^[11]^ using Olink Target genome-wide pQTL mapping.

Pertussis data were sourced from a Finnish database (n = 147, ncontrol = 332,343) (https://storage.googleapis.com/finngen-public-data-r9/summary_stats/finngen_R9_AB1_WHOOPCOUGH.gz).

All included studies received approval from their respective Institutional Review Boards (IRB); thus, reapplication for IRB approval was not required.

### 2.4. Selection of single nucleotide polymorphisms

Initially, SNPs were selected from the summary data of GWAS based on having genome-wide associations with the IVs, significant at *P* < 5 × 10^−8^. However, this criterion yielded a limited number of SNPs. The significance threshold was thus relaxed to 5 × 10^−5^ to prevent false positives and other unreliable results from a small sample size. Subsequently, linkage disequilibrium clumping was applied to exclude unwanted SNPs (*r*^2^ < 0.01, window size = 500 kb; *r*^2^ < 0.001, window size = 10,000 kb).^[12]^ For palindromic SNPs, forward strands were determined using allele frequency. Selected IVs exhibited an *F*-statistic threshold > 10, ensuring that causal estimations were free of weak instrument biases.^[13]^

### 2.5. Statistical analysis

Bidirectional 2-sample MR analysis was conducted using the “2-sample MR” package in R (version 4.3.3). The primary analysis employed the robust IVW meta-analysis.^[8]^ Pleiotropy and outliers were detected using MR-PRESSO, followed by Cochran *Q* test to assess heterogeneity.^[14]^ In the presence of heterogeneity, we opted for a random-effects IVW for the preliminary analysis. Significance was set at *P* < .05. The MR-Egger intercept test was used as a weighted linear regression to evaluate the presence of horizontal pleiotropy in IVs. In addition, a leave-one-out sensitivity test was conducted to assess whether a single SNP significantly influenced the causal effect.

## 3. Results

### 3.1. Causal relationship between immune cells and pertussis

**Figure 2.**
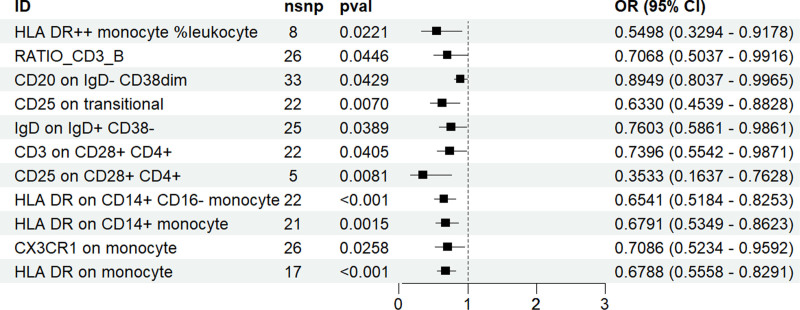
Mendelian randomized analysis of 11 types of immune cells demonstrating a protective effect against pertussis.

In our forward genetic analysis of the relationship between immune cells and pertussis, we identified 30 immune cells with a causal relationship to pertussis. Among these, 11 types of immune cells (HLA-DR++ monocyte %leukocyte, RATIO_CD3_B, CD20 on IgD− CD38dim, CD25 on transitional, IgD on IgD+ CD38−, CD3 on CD28+ CD4+, CD25 on CD28+ CD4+, HLA-DR on CD14+ CD16− monocyte, HLA-DR on CD14+ monocyte, CX3CR1 on monocyte, HLA-DR on monocyte) were found to have a protective effect against pertussis (OR values ranging from 0.3533 to 0.8948), with the strongest protective effect observed in CD25 on CD28+ CD4+ (OR = 0.3533, CI = 0.1636–0.7627, *P* = .008) (Fig. 2).

**Figure 3.**
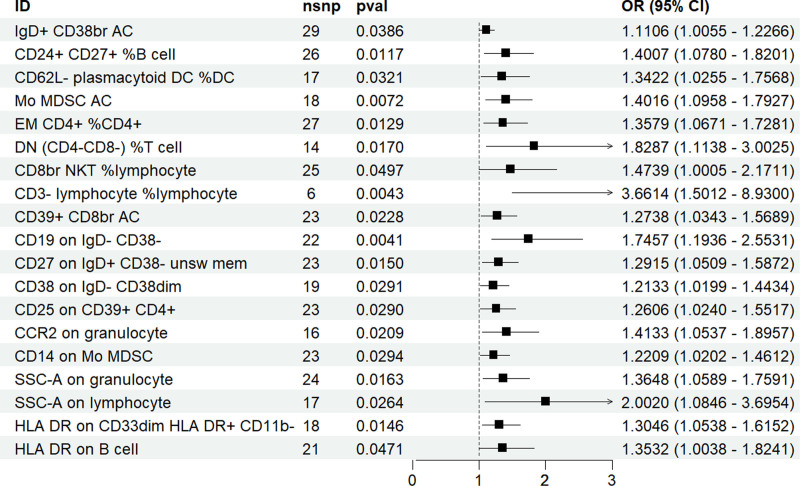
Mendelian randomized analysis of immune cells positively associated with pertussis.

Additionally, 19 types of immune cells were found to increase the risk of pertussis (IgD+ CD38br AC, CD24+ CD27+ %B cell, CD62L− plasmacytoid DC %DC, Mo MDSC AC, EM CD4+ %CD4+, DN (CD4− to CD8−) %T cell, CD8br NKT %lymphocyte, CD3− lymphocyte %lymphocyte, CD39+ CD8br AC, CD19 on IgD− CD38−, CD27 on IgD+ CD38− unsw mem, CD38 on IgD− CD38dim, CD25 on CD39+ CD4+, CCR2 on granulocyte, CD14 on Mo MDSC, SSC-A on granulocyte, SSC-A on lymphocyte, HLA-DR on CD33dim HLA-DR+ CD11b−, HLA-DR on B cell), with the strongest correlation found in CD3− lymphocyte %lymphocyte (OR = 3.6613, CI = 1.5012–8.299, *P* = .0043) (Fig. 3).

Cochran *Q* statistic, the MR-Egger intercept test, and MR-PRESSO indicated no heterogeneity or horizontal pleiotropy in our MR analysis (Table 1). Sensitivity analysis showed that the removal of any single SNP did not significantly affect the causal relationship, indicating the robustness of MR results (Fig. S1, Supplemental Digital Content, http://links.lww.com/MD/O67, and Fig. S2, Supplemental Digital Content, http://links.lww.com/MD/O68).

**Table 1:** Pleiotropy and heterogeneity analyses.

Exposure	Outcome	Pleiotropy		MR-PRESSO		Heterogeneity analyses		
		Egger_intercept	*P*-value	Global test *P*	Correct *P*	Method	*Q*	*P*-value
IgD+ CD38br AC	Pertussis	−0.007	.8798	.506	NA	IVW	27.9373	.4677
CD24+ CD27+ %B cell	Pertussis	−0.0691	.3099	.328	NA	IVW	28.7871	.2729
CD62L− plasmacytoid DC %DC	Pertussis	0.0869	.1815	.832	NA	IVW	11.3215	.7892
Mo MDSC AC	Pertussis	0.0088	.9073	.235	NA	IVW	20.301	.2591
EM CD4+ %CD4+	Pertussis	−0.0241	.649	.863	NA	IVW	19.4022	.819
DN (CD4−CD8−) %T cell	Pertussis	−0.0799	.451	.563	NA	IVW	11.8641	.5388
CD8br NKT %lymphocyte	Pertussis	0.0711	.3347	.588	NA	IVW	22.3801	.5565
CD3− lymphocyte %lymphocyte	Pertussis	−0.0156	.9642	.424	NA	IVW	5.4049	.3684
CD39+ CD8br AC	Pertussis	−0.1025	.0962	.573	NA	IVW	19.6264	.6063
CD19 on IgD− CD38−	Pertussis	0.0498	.4492	.447	NA	IVW	21.8064	.4107
CD27 on IgD+ CD38− unsw mem	Pertussis	0.0390	.6366	.954	NA	IVW	12.6161	.9433
CD38 on IgD− CD38dim	Pertussis	0.0097	.8835	.977	NA	IVW	8.5871	.9685
CD25 on CD39+ CD4+	Pertussis	0.0523	.4817	.836	NA	IVW	16.4152	.7947
CCR2 on granulocyte	Pertussis	0.0385	.5581	.546	NA	IVW	14.6512	.4768
CD14 on Mo MDSC	Pertussis	−0.0089	.8818	.822	NA	IVW	16.9843	.7642
SSC-A on granulocyte	Pertussis	0.0711	.2755	.273	NA	IVW	26.0432	.2988
SSC-A on lymphocyte	Pertussis	0.3286	.0563	.082	NA	IVW	24.0458	.0885
HLA-DR on CD33dim HLA-DR+ CD11b-	Pertussis	−0.0653	.3737	.721	NA	IVW	13.5526	.6984
HLA-DR on B cell	Pertussis	0.0317	.7615	.120	NA	IVW	42.4486	.0024
HLA-DR++ monocyte %leukocyte	Pertussis	−0.1133	.4359	.222	NA	IVW	9.5688	.2143
RATIO_CD3_B	Pertussis	−0.0016	.9805	.158	NA	IVW	32.1368	.1540
CD20 on IgD− CD38dim	Pertussis	−0.0673	.0882	.168	NA	IVW	44.7383	.0667
CD25 on transitional	Pertussis	0.0580	.3826	.879	NA	IVW	13.8088	.8776
IgD on IgD+ CD38−	Pertussis	−0.0806	.1952	.269	NA	IVW	27.7039	.2727
CD3 on CD28+ CD4+	Pertussis	0.0355	.642	.578	NA	IVW	20.0345	.5190
CD25 on CD28+ CD4+	Pertussis	−0.3473	.1766	.489	NA	IVW	4.2008	.3795
HLA-DR on CD14+ CD16− monocyte	Pertussis	0.1043	.2133	.696	NA	IVW	17.8331	.6595
HLA-DR on CD14+ monocyte	Pertussis	0.1273	.1369	.841	NA	IVW	13.0447	.8754
CX3CR1 on monocyte	Pertussis	0.1167	.1514	.354	NA	IVW	27.6105	.3260
HLA-DR on monocyte	Pertussis	−0.0625	.4471	.777	NA	IVW	10.5737	.8350

To check for potential reverse causality, we conducted a reverse MR analysis (with pertussis as the exposure and the 30 immune cells with causal relationships as the outcomes). The results indicated that pertussis had no effect on these 30 immune cells, as all *P*-values were >.05 (Fig. S3, Supplemental Digital Content, http://links.lww.com/MD/O69).

### 3.2. Relationship between inflammatory cytokines and pertussis

Three inflammatory cytokines were found to have a causal relationship with pertussis: IL-4 (OR = 0.3688, 95% CI = 0.16571–0.8208, *P* = .0145418), IL-18R1 (OR = 1.2757, 95% CI = 1.0499–1.5501, *P* = .0143), and FGF-21 (OR = 2.0747, 95% CI = 1.0669–4.0341, *P* = .03147).

### 3.3. Mediating role of inflammatory cytokines in the causal relationship between immune cells and pertussis

We established 8 pairs of mediation relationships, involving 8 immune cells and 3 inflammatory cytokines. Our analysis showed that none of these 8 mediation relationships had statistical significance, as all *P*-values were >.05 (Table 2).

**Table 2:** Intermediate analysis result.

Exposure (immune cell)	Mediating factors (inflammatory cytokines)	Outcome	se	*Z*	Mediation effects(95%CI)	*P*-value
CD27 on IgD+ CD38− unsw mem	IL-18R1	Pertussis	0.0242	0.219	0.0053 (−0.0421, 0.0527)	.8266
SSC-A on granulocyte	IL-18R1	Pertussis	0.0242	0.3064	0.0074 (−0.04, 0.0549)	.7593
IgD+ CD38br AC	FGF-21	Pertussis	0.2476	0.0764	0.0189 (−0.4664, 0.5042)	.9391
CD25 on transitional	IL-4	Pertussis	0.4071	0.1028	0.0418 (−0.7561,0.8398)	.9181
CD27 on IgD+ CD38− unsw mem	IL-4	Pertussis	0.4071	0.0684	0.0278 (−0.7702, 0.8258)	.9455
HLA-DR on CD14+ CD16− monocyte	IL-4	Pertussis	0.4071	−0.1105	−0.045 (−0.843, 0.753)	.912
HLA-DR on CD14+ monocyte	IL-4	Pertussis	0.4071	−0.1062	−0.0431 (−0.8412, 0.7548)	.9154
HLA-DR on monocyte	IL-4	Pertussis	0.4071	−0.0733	−0.03 (−0.8412, 0.7681	.9416

## 4. Discussion

This study employed a bidirectional 2-sample MR analysis to explore the causal relationships between immune cells and inflammatory cytokines in pertussis and provided large-scale genetic data support for the first time. The results indicate that specific immune cell types have a significant causal relationship with pertussis, while inflammatory cytokines, although associated with pertussis, do not mediate the effect of immune cells on the disease. These findings offer new insights into the immunological mechanisms of pertussis and may provide a basis for future prevention and treatment strategies.

### 4.1. Protective role of immune cells against pertussis

In this study, 11 types of immune cells exhibited protective effects against pertussis, highlighting their crucial role in combating the infection. The most significant protective effect was observed in CD25 on CD28+ CD4+ cells, a subset that plays a key role in regulating and maintaining immune system balance.^[15]^ CD4+ T cells are central to the immune system, responsible for coordinating immune responses, including activating other immune cells such as B cells and macrophages, as well as directly killing infected cells.^[16]^ CD25 (IL-2 receptor α chain) is highly expressed on activated T cells, enhancing their proliferation and survival, thus boosting infection resistance.^[17]^

HLA-DR+ monocytes also showed significant protective effects in this study (including HLA-DR on CD14+ CD16− monocytes, HLA-DR on CD14+ monocytes, and HLA-DR on monocytes). HLA-DR is a major histocompatibility complex class II molecule involved in antigen presentation, promoting T cell activation and immune response.^[18]^ By expressing HLA-DR, these monocytes can effectively recognize and present pathogen antigens, triggering specific immune responses to prevent the spread of pertussis.^[19]^

Additionally, CD25 on transitional B cells and CD3 on CD28+ CD4+ T cells play important roles in protecting against pertussis. Transitional B cells are intermediate stages between immature and mature B cells, playing a crucial role in antibody production and immune memory formation.^[20]^ CD28 is a co-stimulatory molecule on T cells that works with the CD3 complex to enhance T cell activation and survival, thereby strengthening immune response.^[21]^

### 4.2. Immune cells increasing the risk of pertussis

Conversely, this study found 19 types of immune cells associated with an increased risk of pertussis, with the strongest correlation seen in CD3− lymphocyte %lymphocyte. These cell subsets might promote the growth and spread of pathogens under certain conditions.^[22]^ For example, CD3− lymphocytes may represent a group of immature or dysfunctional immune cells that are ineffective in combating the pertussis pathogen.^[23]^ An increase in these cells could reflect a state of immune system dysregulation, leading to weakened immune defense against pathogens.

Other immune cells associated with increased pertussis risk include IgD+ CD38br AC and CD24+ CD27+ %B cells. IgD+ CD38br AC cells are a type of activated B cells, often elevated in chronic inflammation or autoimmune diseases.^[23]^ Overactivation of these cells may result in an excessive immune response, disrupting normal immune balance and increasing infection risk.^[24]^ CD24+ CD27+ %B cells are a type of memory B cell, typically involved in secondary immune responses.^[25]^ However, an excess of memory B cells may lead to abnormal immune reactions, raising the risk of infection.

### 4.3. The role of inflammatory cytokines in immune responses

This study also identified 3 inflammatory cytokines (IL-4, IL-18R1, and FGF-21) with causal relationships with pertussis, suggesting that these cytokines may play direct roles in the development of the disease. IL-4, a classical Th2 cytokine, is typically associated with humoral immune responses and antibody production. IL-4 is a key anti-inflammatory cytokine primarily produced by Th2 helper T cells. It plays a central role in regulating immune responses, promoting B cell proliferation and differentiation, enhancing antibody production, and inhibiting Th1-type immune responses.^[26]^ Its causal role in pertussis may be related to its promotion of antibody generation and regulation of immune balance.^[27]^ The findings related to IL-18R1 and FGF-21 are novel, particularly IL-18R1, IL-18R1 is the receptor for interleukin-18 (IL-18), an important pro-inflammatory cytokine involved in the immune system’s response to pathogens. IL-18R1 is expressed on various immune cells, such as natural killer cells, T cells, and monocytes, and plays a crucial role in regulating the immune response to pathogens like viruses and bacteria. Activation of IL-18R1 also enhances the production of other pro-inflammatory cytokines, amplifying the inflammatory response. While this is beneficial in combating bacterial and viral infections, excessive activation can lead to tissue damage, especially in severe infections (e.g., pneumonia) or cytokine storms..^[28]^ FGF-21, as a metabolic regulator, may promote pertussis indirectly by influencing immune metabolism.^[29]^

### 4.4. The non-mediating role of inflammatory cytokines

Although IL-4, IL-18R1, and FGF-21 were found to have direct causal relationships with pertussis, the 2-step MR analysis showed that inflammatory cytokines do not mediate the effect of immune cells on pertussis. This result was unexpected, particularly given that inflammatory cytokines are commonly key mediators in many immune-mediated diseases.^[30,31]^ A possible explanation is that while inflammatory cytokines directly affect pertussis, their roles may not be directly related to the function of immune cells, or their regulatory roles are too complex to be fully captured by the current model.

### 4.5. Study limitations

Despite providing important findings through large-scale GWAS data, this study has certain limitations. First, MR methods rely on the strength of genetic instruments, which cannot entirely eliminate potential biases. Second, while we established causal relationships between immune cells and pertussis, the underlying biological mechanisms remain to be fully elucidated and require further experimental validation. Third, the complex roles of inflammatory cytokines may vary in different immune states, and future studies will need to validate these cytokines in various infection contexts. Finally, the results of this study are primarily based on genetic data from European populations, so their generalizability to other ethnicities or geographic regions remains unclear. Therefore, future research needs to validate these findings in more diverse populations across different ethnicities and regions to further clarify the universal causal relationships between immune cells, inflammatory cytokines, and pertussis.

### 4.6. Prospects and future research directions

This study provides important preliminary insights into the immunological mechanisms of pertussis, particularly regarding the causal relationship between immune cells and pertussis. However, future research should further explore the roles of these immune cells and inflammatory factors in different populations, especially examining how factors like age or varying immune statuses (e.g., children, the elderly, and immunosuppressed patients) affect the immune response to pertussis. Given the global public health burden of pertussis, future studies should focus on cross-ethnic and cross-regional populations to better understand the impact of immunogenetic backgrounds on pertussis susceptibility.

While inflammatory cytokines have not been confirmed as mediators between immune cells and pertussis in this study, future research could investigate other potential mediators not covered here, or as-yet-undefined signaling pathways. This may reveal new regulatory mechanisms of pertussis pathogenesis, thereby providing a theoretical basis for developing new preventive and therapeutic strategies.

## Acknowledgments

We would like to thank all the authors who contributed to the drafting of the manuscript.

## Author contributions

**Conceptualization:** Fu-Qing Lu, Ji-Gan Wang, Kun-Ling Song.

**Formal analysis:** Ji-Gan Wang, Kun-Ling Song.

**Investigation:** Hui-Mei Feng, Kun-Ling Song.

**Methodology:** Hui-Mei Feng.

**Software:** Kun-Ling Song.

**Supervision:** Fu-Qing Lu.

**Writing – original draft:** Fu-Qing Lu.

**Writing – review & editing:** Hui-Mei Feng.

## Supplementary Material


