# Supplementary figures and images for: Causal relationships between immune cells, inflammatory cytokines, and pertussis: Bidirectional 2-sample Mendelian randomization study and mediation analysis
Source: Medicine (Baltimore). 2024 Nov 29;103(48):e40712. doi: 10.1097/MD.0000000000040712 (PMC11608674; doi:10.1097/MD.0000000000040712)

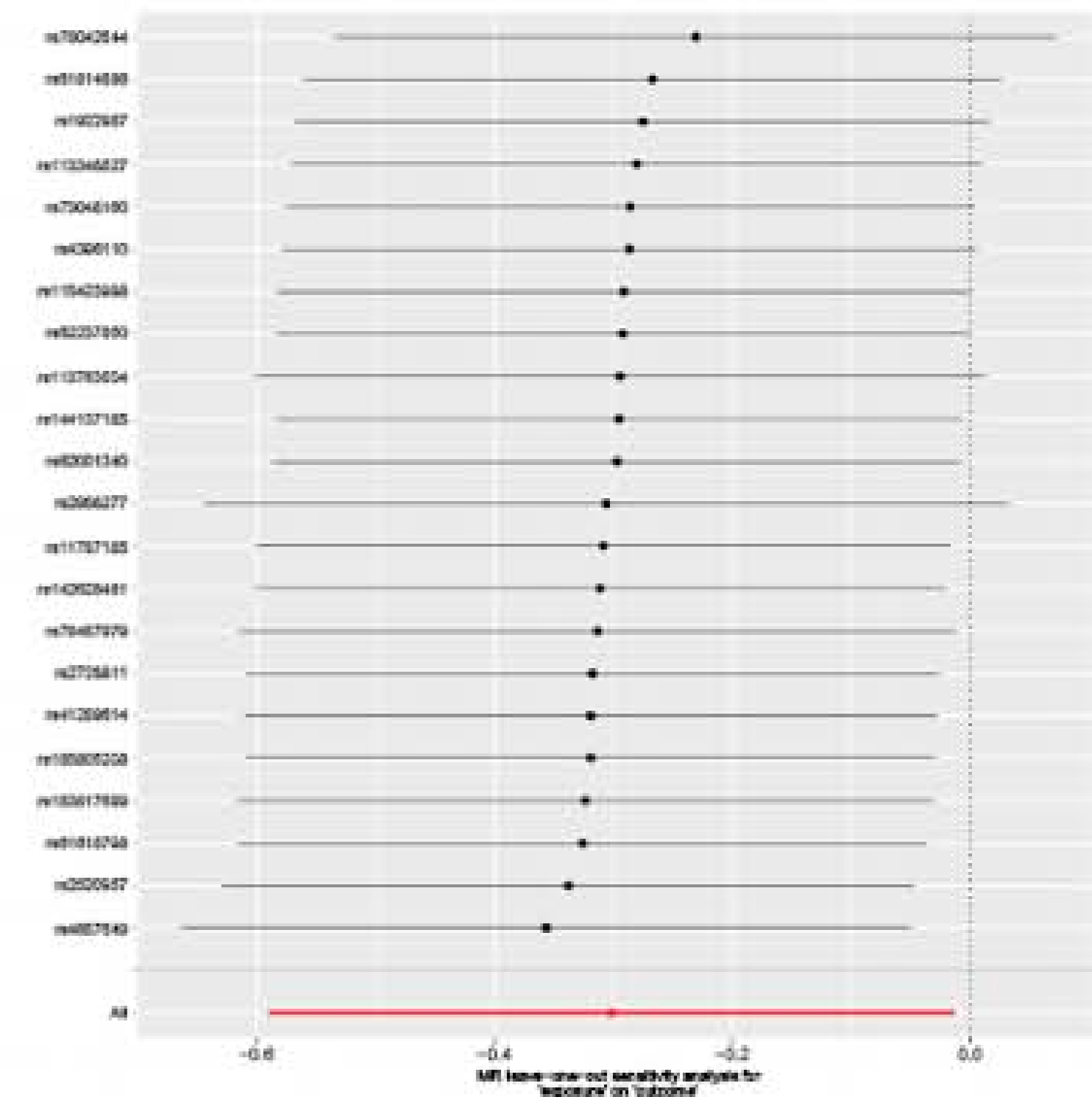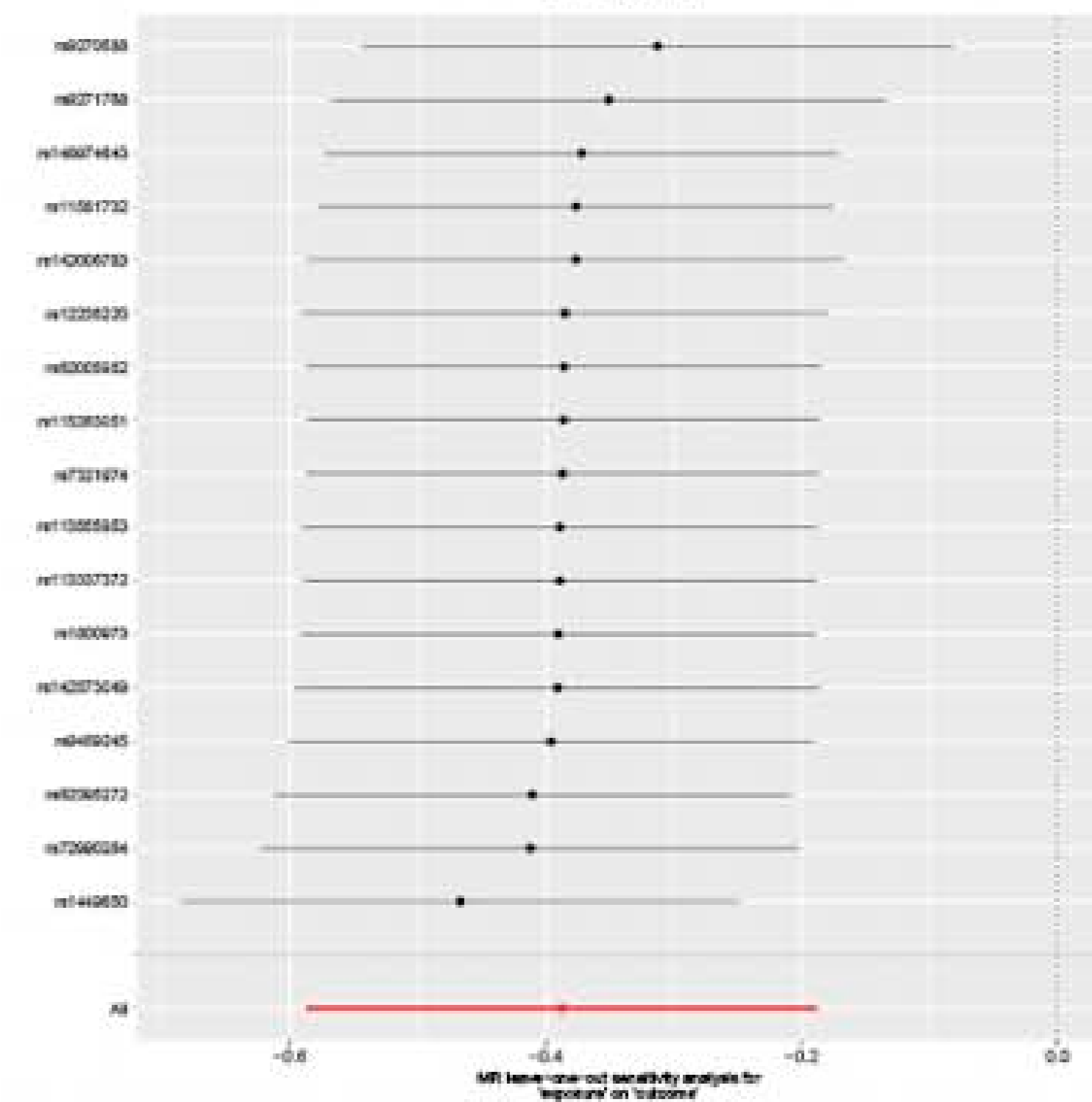

Supplement: Supplementary file 2 [file medi-103-e40712-s002.pdf]

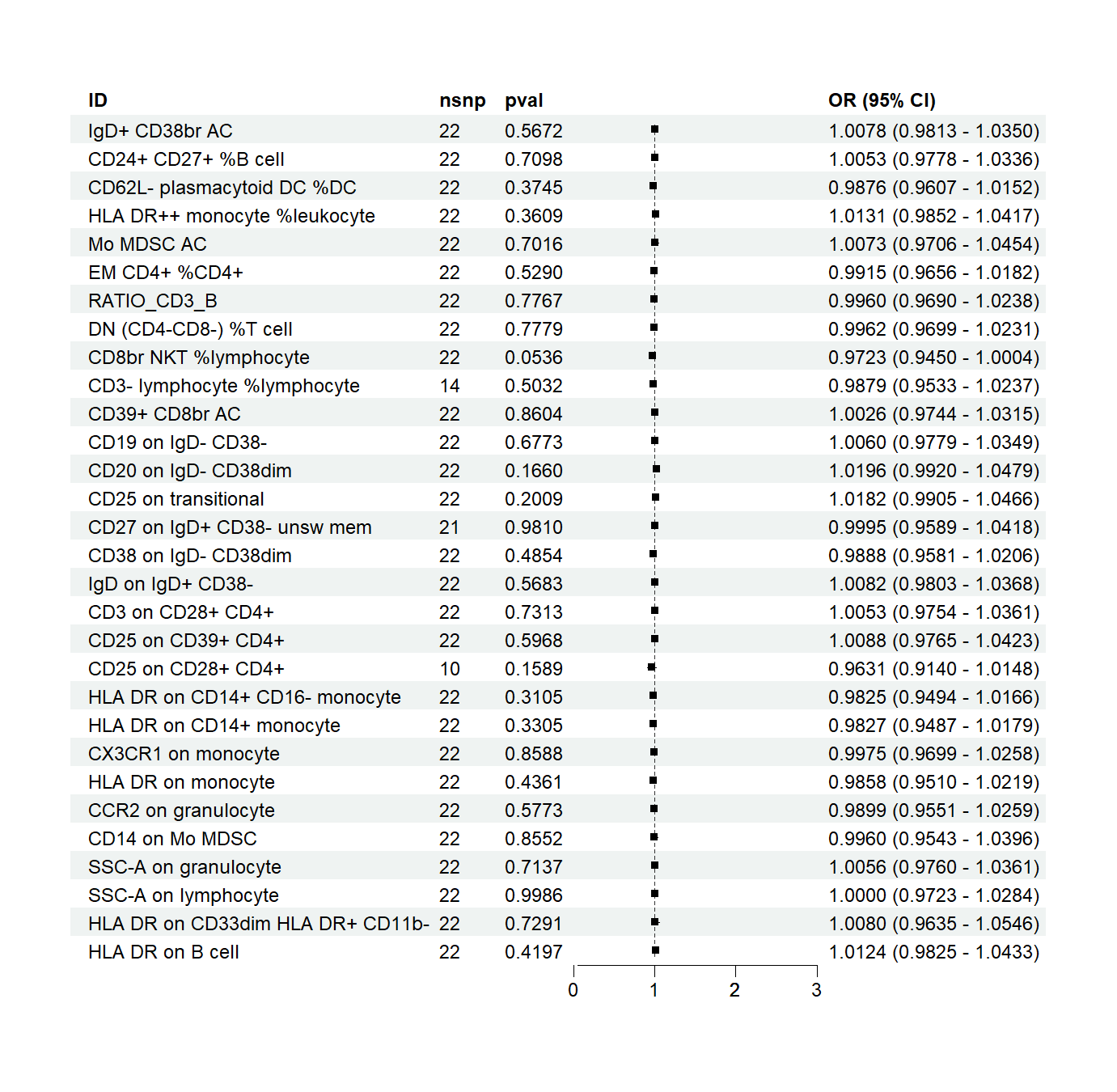

Supplement: Supplementary file 4 [file medi-103-e40712-s004.tiff]
