# Supplementary material for: Causal relationships between immune cells, inflammatory cytokines, and pertussis: Bidirectional 2-sample Mendelian randomization study and mediation analysis
Source: Medicine (Baltimore). 2024 Nov 29;103(48):e40712. doi: 10.1097/MD.0000000000040712 (PMC11608674; doi:10.1097/MD.0000000000040712)
Supplement: Supplementary file 3 [file medi-103-e40712-s003.pdf]

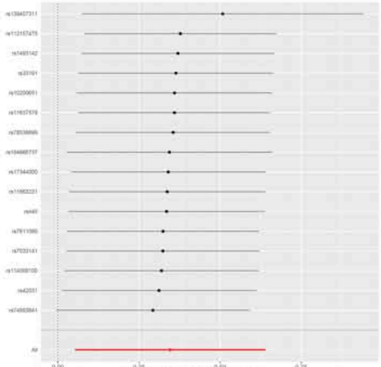
CCR2 on granulocyte.pdf

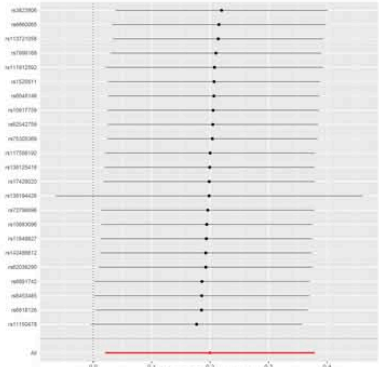
CD14 on Mo MDSC.pdf

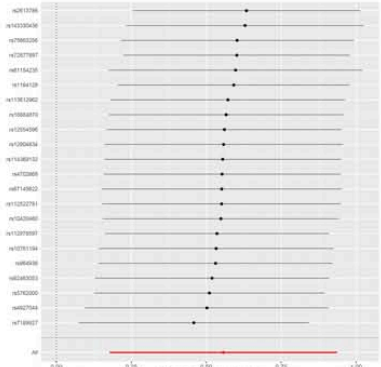
CD19 on IgD- CD38-.pdf

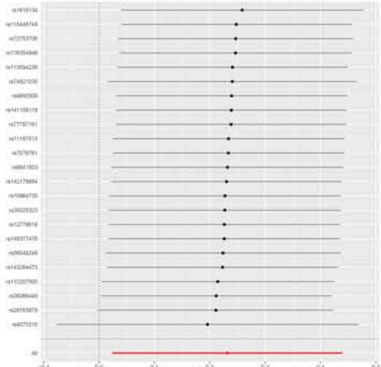
CD25 on CD39+ CD4+.pdf

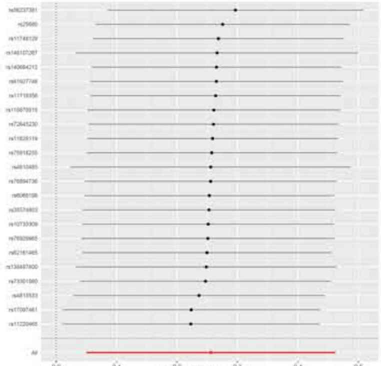
CD27 on IgD+ CD38- unsw mem.pdf

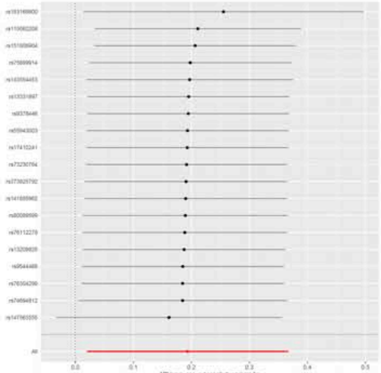
CD38 on IgD- CD38dim.pdf

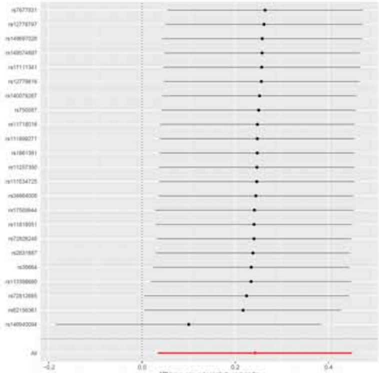
CD39+ CD8br AC.pdf

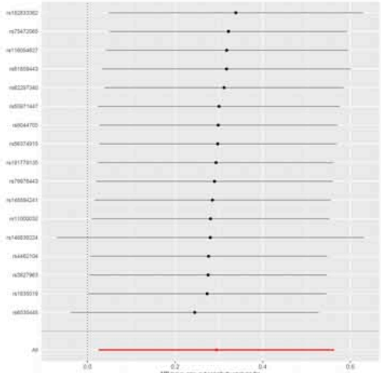
CD62L- plasmacytoid DC %DC.pdf

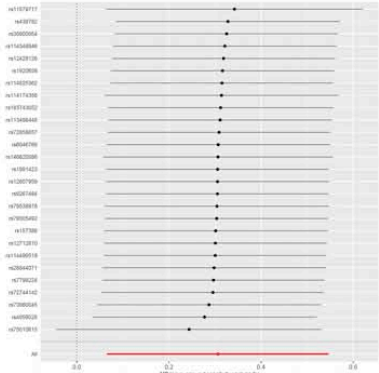
EM CD4+ %CD4+.pdf

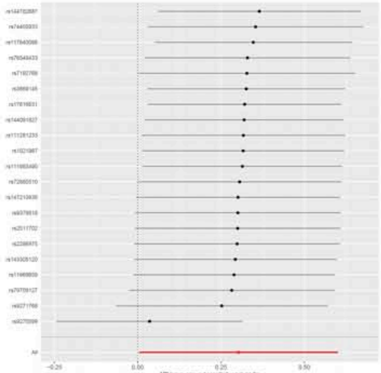
HLA DR on B cell.pdf

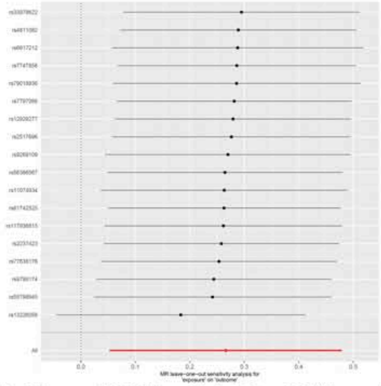
HLA DR on CD33dim HLA DR+ CD11b-.pdf

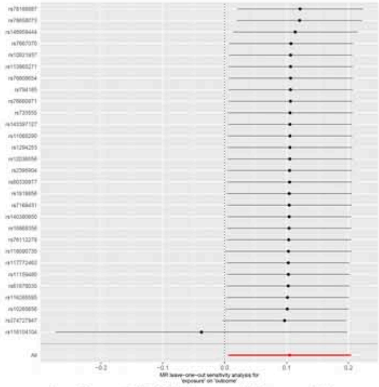
IgD+ CD38br AC.pdf

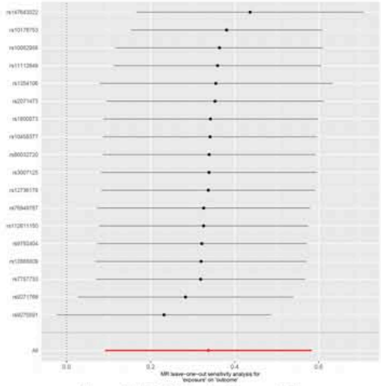
Mo MDSC AC.pdf

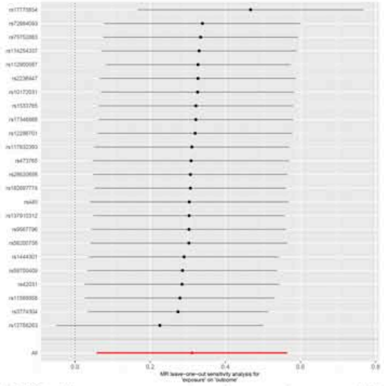
SSC-A on granulocyte.pdf

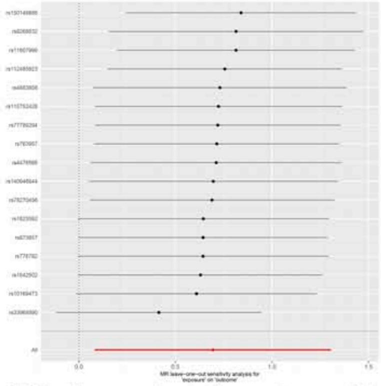
SSC-A on lymphocyte.pdf

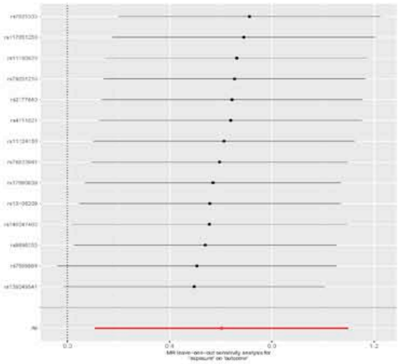
DN (CD4-CD8-) %T cell

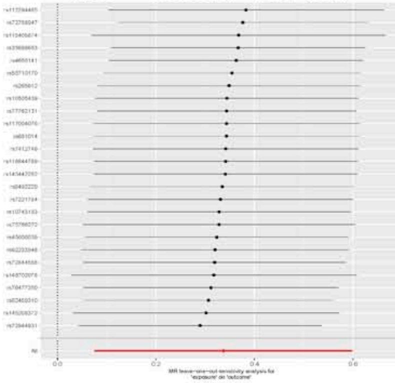
CD24+ CD27+ %B cell

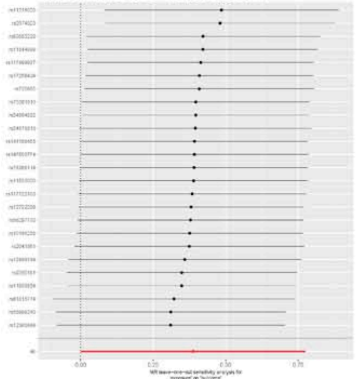
CD8br NKT %lymphocyte

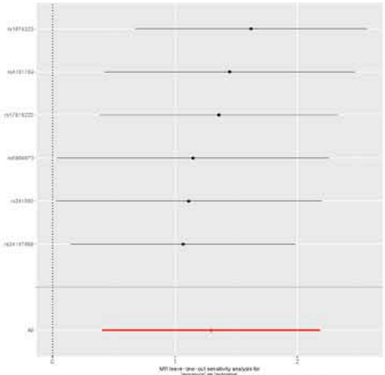
CD3- lymphocyte %lymphocyte
